# Common and distinct patterns of grey-matter volume alteration in major depression and bipolar disorder: evidence from voxel-based meta-analysis

**DOI:** 10.1038/mp.2016.72

**Published:** 2016-05-24

**Authors:** T Wise, J Radua, E Via, N Cardoner, O Abe, T M Adams, F Amico, Y Cheng, J H Cole, C de Azevedo Marques Périco, D P Dickstein, T F D Farrow, T Frodl, G Wagner, I H Gotlib, O Gruber, B J Ham, D E Job, M J Kempton, M J Kim, P C M P Koolschijn, G S Malhi, D Mataix-Cols, A M McIntosh, A C Nugent, J T O'Brien, S Pezzoli, M L Phillips, P S Sachdev, G Salvadore, S Selvaraj, A C Stanfield, A J Thomas, M J van Tol, N J A van der Wee, D J Veltman, A H Young, C H Fu, A J Cleare, D Arnone

**Affiliations:** 1 Department of Psychological Medicine, Centre for Affective Disorders, Institute of Psychiatry, Psychology and Neuroscience, King’s College London, London, UK; 2 Department of Psychosis Studies, Institute of Psychiatry, Psychology and Neuroscience, King’s College London, London, UK; 3 Research Unit, FIDMAG Germanes Hospitalàries – CIBERSAM, Sant Boi de Llobregat, Barcelona, Spain; 4 Department of Clinical Neuroscience, Karolinska Institutet, Stockholm, Sweden; 5 Mental Health, Parc Taulí Sabadell-CIBERSAM, University Hospital, Sabadell, Barcelona, Spain; 6 Department of Psychiatry, Bellvitge University Hospital-IDIBELL, Barcelona, Spain; 7 Department of Radiology, Nihon University School of Medicine, Tokyo, Japan; 8 Department of Psychiatry, Trinity College Institute of Neuroscience, Trinity College, Dublin, Ireland; 9 Department of Psychiatry, The 1st Affiliated Hospital of Kunming Medical University, Kunming, PR China; 10 Computational, Cognitive and Clinical Neuroimaging Laboratory, Department of Medicine, Imperial College London, London, UK; 11 Department of Neuroscience, Medical School, Fundação do ABC, Santo André, SP, Brazil; 12 ABC Center of Studies on Mental Health, Santo André, SP, Brazil; 13 PediMIND Program, Bradley Hospital, Department of Psychiatry, Brown University, East Providence, RI, USA; 14 Academic Clinical Neurology, Department of Neuroscience, University of Sheffield, Sheffield, UK; 15 Department of Psychiatry and Psychotherapy, Otto-von-Guericke University, Magdeburg, Germany; 16 Department of Psychiatry, University of Dublin, Trinity College, Dublin, Ireland; 17 Psychiatric Brain and Body Research Group Jena, Department of Psychiatry and Psychotherapy, Jena University Hospital, Jena, Germany; 18 Department of Psychology, Stanford University, Stanford, CA, USA; 19 Section for Experimental Psychopathology and Neuroimaging, Department of General Psychiatry, Heidelberg University Hospital, Heidelberg, Germany; 20 Department of Psychiatry, Korea University College of Medicine, Seoul, South Korea; 21 Neuroimaging Sciences, University of Edinburgh, Edinburgh, UK; 22 Scottish Imaging Network–A Platform for Scientific Excellence (SINAPSE), Giffnock, UK; 23 Centre for Clinical Brain Sciences, University of Edinburgh, Edinburgh, UK; 24 Department of Neuroimaging, Institute of Psychiatry, Psychology and Neuroscience, King’s College London, London, UK; 25 Department of Psychological and Brain Sciences, Dartmouth College, Hanover, NH, USA; 26 Department of Psychology, Dutch Autism and ADHD Research Center, Brain and Cognition, University of Amsterdam, Amsterdam, The Netherlands; 27 CADE Clinic, Discipline of Psychiatry, Sydney Medical School, University of Sydney, Sydney, NSW, Australia; 28 Division of Psychiatry, University of Edinburgh, Edinburgh, UK; 29 Experimental Therapeutics and Pathophysiology Branch, Intramural Research Program, National Institute of Mental Health, National Institutes of Health, Bethesda, MD, USA; 30 Institute for Ageing and Health, Newcastle University, Campus for Ageing and Vitality, Newcastle upon Tyne, UK; 31 Department of Psychiatry, University of Cambridge, Cambridge, UK; 32 Department of Neuroscience, Medical School, University of Sheffield, Sheffield, UK; 33 Department of Psychiatry, University of Pittsburgh, School of Medicine, Pittsburgh, PA; 34 Centre for Healthy Brain Ageing (CHeBA), School of Psychiatry, University of New South Wales, Randwick, NSW, Australia; 35 Neuropsychiatric Institute, Prince of Wales Hospital, Randwick, NSW, Australia; 36 Janssen Research and Development, Titusville, NJ, USA; 37 Department of Psychiatry and Behavioral Sciences, Center of Excellence on Mood Disorders, Medical School, University of Texas Health Science Center at Houston, Houston, TX, USA; 38 The Patrick Wild Centre, Royal Edinburgh Hospital, University of Edinburgh, Edinburgh, UK; 39 NeuroImaging Center, University Medical Center Groningen, University of Groningen, Groningen, The Netherlands; 40 Department of Psychiatry, Leiden University Medical Center, Leiden University, Leiden, The Netherlands; 41 Leiden Institute for Brain and Cognition, Leiden, The Netherlands; 42 Department of Psychiatry, VU University Medical Center, Amsterdam, The Netherlands; 43 School of Psychology, University of East London, London, UK

## Abstract

Finding robust brain substrates of mood disorders is an important target for research. The degree to which major depression (MDD) and bipolar disorder (BD) are associated with common and/or distinct patterns of volumetric changes is nevertheless unclear. Furthermore, the extant literature is heterogeneous with respect to the nature of these changes. We report a meta-analysis of voxel-based morphometry (VBM) studies in MDD and BD. We identified studies published up to January 2015 that compared grey matter in MDD (50 data sets including 4101 individuals) and BD (36 data sets including 2407 individuals) using whole-brain VBM. We used statistical maps from the studies included where available and reported peak coordinates otherwise. Group comparisons and conjunction analyses identified regions in which the disorders showed common and distinct patterns of volumetric alteration. Both disorders were associated with lower grey-matter volume relative to healthy individuals in a number of areas. Conjunction analysis showed smaller volumes in both disorders in clusters in the dorsomedial and ventromedial prefrontal cortex, including the anterior cingulate cortex and bilateral insula. Group comparisons indicated that findings of smaller grey-matter volumes relative to controls in the right dorsolateral prefrontal cortex and left hippocampus, along with cerebellar, temporal and parietal regions were more substantial in major depression. These results suggest that MDD and BD are characterised by both common and distinct patterns of grey-matter volume changes. This combination of differences and similarities has the potential to inform the development of diagnostic biomarkers for these conditions.

## Introduction

Affective disorders such as major depression (MDD) and bipolar disorder (BD) are serious conditions that significantly affect quality of life.^1, 2^ In the absence of a definitive understanding of the neuropathology underpinning these disorders, no clinical biomarkers are currently available to aid diagnosis and treatment.^3, 4, 5^ This is a particularly significant issue given the frequency of misdiagnosis and inappropriate treatment in affective disorders.^6^ As a result, biomarker discovery and optimisation are essential steps for future progress.

Neuroimaging studies have identified a number of differences between patients with affective disorders and healthy individuals in brain volume, function, neurochemistry and connectivity in key neurobiological circuits involved in mood regulation.^3, 7, 8, 9, 10, 11^ Grey-matter volume changes in affective disorders have been well documented in a number of cortical and subcortical structures including prefrontal regions and the hippocampus.^10, 12, 13, 14^ It is at present unclear to what extent specific or common morphological alterations occur in MDD and BDs given the paucity of direct comparisons and inconsistencies in the available findings. The two studies that have addressed this issue have identified differences in prefrontal regions; however, the precise location differs in these studies.^15, 16^ Gaining a more detailed insight into the neuropathological relationship between these disorders is an essential step in providing a more precise definition of candidate diagnostic biomarkers at the brain level, which could improve current classifications of affective disorders.

The aim of this meta-analysis was to use the largest database of voxel-based morphometry (VBM) studies in affective disorders to date by taking advantage of a thorough and detailed meta-analytic technique to (1) identify morphometric changes in MDD and BD compared with healthy controls and (2) compare results across diagnostic groups to assess morphometric differences and similarities that may reflect common and/or distinct neuropathological pathways in affective disorders. Most importantly, we adopt an improved meta-analytic technique with increased sensitivity, specificity and reliability of the analyses, by combining statistical maps from some of the original studies with peak coordinates conventionally used in neuroimaging meta-analyses.

## Materials and methods

### Literature searches

We searched Pubmed, Scopus and ScienceDirect for studies comparing patients with MDD or BD with control groups published up to January 2015 using the following keywords: Magnetic resonance imaging OR MRI AND depression OR BD OR mania OR mood disorders. Broad search terms were used to minimise the likelihood of missing any relevant studies. Reviews and meta-analyses were cross-referenced to identify studies that were missed in the literature searches. Authors were contacted for unpublished data including t-maps from the original studies. A systematic approach compliant with PRISMA (Preferred Reporting Items for Systematic Reviews and Meta-Analyses) guidelines^17^ was adopted.

Studies were excluded if: (1) they adopted neuroimaging techniques other than MRI whole-brain VBM; (2) participants age was below 18 or above 65 (to minimise the effect of neurodevelopment and neurodegeneration, respectively); (3) samples were confounded by any comorbid neurological conditions; (4) t-maps were unavailable, consistent statistical thresholds throughout the brain were not used or peak coordinates were not reported; and (5) included ⩽10 patients. If the same patient group was used in multiple studies, then only the study with the largest sample was included. Conversely, when the same control group was used in several subgroup comparisons, only a combined summary result was included in the meta-analysis. For studies that used longitudinal treatment designs, only baseline pre-treatment data were included.

### Meta-analysis

Meta-analysis was performed using the anisotropic effect size version of Seed-based *D* Mapping (formerly Signed Differential Mapping, AES-SDM).^18^ This method has been described in detail elsewhere.^19, 20^ In summary, AES-SDM allows combination of peak coordinates and statistical parametric maps to create whole-brain effect size and variance maps, which are then used to perform voxel-wise random effects meta-analyses. Although meta-analyses based on peak coordinates alone are able to produce reliable results, the addition of original statistical maps substantially improves the sensitivity of the analyses.^19^ AES-SDM allows meta-analytic summaries of grey-matter volumes within each disorder (for example, MDD and BD vs healthy controls) and comparisons of abnormalities between conditions (for example, MDD vs BD) based on the evaluation of effect sizes. Finally, the multimodal analysis function of the AES-SDM statistical package allows conjunction analyses to be performed, which enabled us to identify regions where both patient groups show common differences with respect to controls, while taking into account error in the estimation of the magnitude of these differences.^21^

AES-SDM also allows heterogeneity to be systematically quantified in a voxel-wise manner using the Q statistic. The overlap between significant areas of heterogeneity with areas of grey-matter differences was systematically investigated with separate simple meta-regressions using available potential confounders where these were provided in a sufficient proportion of the included studies. Given the relatively small number of studies, we set the cutoff for inclusion of potential confounders in meta-regressions to ⩾20 studies in order to minimise the occurrence of false positives.^22^ For MDD, we conducted meta-regressions with antidepressant medication use, depression duration (from age of onset), depression severity, mean patient age and sex. For BD, we used mood state, depression duration, antipsychotic medication use, lithium use, mean patient age and sex. We also examined effects of magnetic field strength and image smoothing level for both conditions. Studies that did not report these measures were excluded from these analyses. To enable meta-regressions to be conducted using a consistent measure of depression severity, when studies reported Montgomery-Åsberg Depressing Rating Scale^23^ scores, these were converted to Hamilton Depression Rating Scale^24^ equivalents using the method devised by Heo *et al.*^25^ Group differences in demographic and clinical variables were explored to discover any potential confounders in group comparisons by using standard meta-analytic tests weighted by sample size.

Significant clusters were tested for publication bias using funnel plots and Egger’s test on effect size estimates extracted from the cluster peak, performed using the Metafor package^26^ for R (http://www.rproject.org). Funnel plots of effect sizes in each cluster were also visually inspected to ensure that results were robust. Finally, we assessed reliability of our meta-analytic results with a jack-knife analysis, in which the meta-analysis is rerun iteratively with each study left out in one iteration. This method assesses the reliability of significant results, on the assumption that reliable results should be robust to the removal of individual studies, and should therefore remain present in the majority of jack-knife iterations. Clusters that were no longer significant in the meta-analysis in 10% or more of the iterations were rejected as we wished to include the most robust results, which should be present in the majority of jack-knife iterations. In the text, we only report clusters that met our criteria for robustness. We provide full results and details regarding the meta-analysis method in Supplementary methods.

## Results

### Literature searches

Literature searches produced 14 951 results, of which 66 studies met criteria for inclusion (Supplementary Figure 1). We also identified five studies from previous meta-analyses and reviews. In addition, we had access to statistical maps from two unpublished studies, one in MDD and the other in BD. This resulted in a total of 73 studies included in the final analysis. Details are presented in Supplementary Tables 1 and 2.

### Study characteristics

#### Major depression

We identified 41 studies that included 50 comparisons between patients and healthy controls (Supplementary Table 1), of which statistical maps were available for 9. These studies included a total of 1736 patients and 2365 healthy controls. Patients’ mean age was 38.5 years (s.d.=9.7) and 38.7% were male. The mean age of healthy control participants was 37.1 years (s.d.=7.8), and 39.5% were male. In studies that provided information on mood state, 1348 patients (94%) experienced symptoms of depression at the time of scanning and 88 (6%) were euthymic.

Demographic details were well reported across studies (41 studies, 98%). With regard to clinical information, 11 studies (26%) did not mention depression severity and 7 (17%) did not refer to duration of illness.

#### Bipolar disorder

We identified 32 studies with 36 comparisons between patients and healthy controls (Supplementary Table 2), representing a total of 980 patients and 1427 controls. Original statistical maps were available for six of these studies. Demographic details were reported in all studies. Availability of clinical information was less consistent with 8 studies (25%) not reporting the number of medicated patients, and 11 (34%) not providing treatment details. With regard to symptoms, 9 (28%) studies did not report the mood state, 17 studies (53%) did not provide a measure of depressive symptoms, 16 studies (50%) did not provide information on manic symptoms, 3 studies (9%) did not provide a measure of illness duration and 18 (56%) did not provide information on symptoms severity. The mean age of patients was 37.6 years (s.d.=4.4) and 44.1% were male. The mean age of controls was 35.9 years (s.d.=4.8), and 43.8% were male. In relation to sub-types, 808 patients (82%) experienced type I disorder, 91 (9%) were diagnosed as type II and for 81 patients (8%) this information was not available. In the studies that provided details of mood state, 438 patients (56%) were euthymic at the time of scanning, 218 (28%) experienced symptoms of depression, 118 (15%) were manic and 5 (1%) had mixed symptoms.

#### MDD vs BD

Studies in MDD and BD included patients of similar ages (omnibus test *Q*_M_^(1)^=0.28, *P*=0.60) and sex (*Q*_M_^(1)^=0.95, *P*=0.33). Studies that reported duration of illness suggested shorter total durations of illness in patients with MDD than BD (weighted means 8.1 vs 12.5 years, *Q*_M_^(1)^=9.51, *P*⩽0.002). Predictably, more patients were in a depressive state at the time of scanning in MDD studies (*χ*^2^_(1)_=22.30, *P*<0.001).

### Meta-analysis

#### MDD vs healthy controls

**Figure 1:**
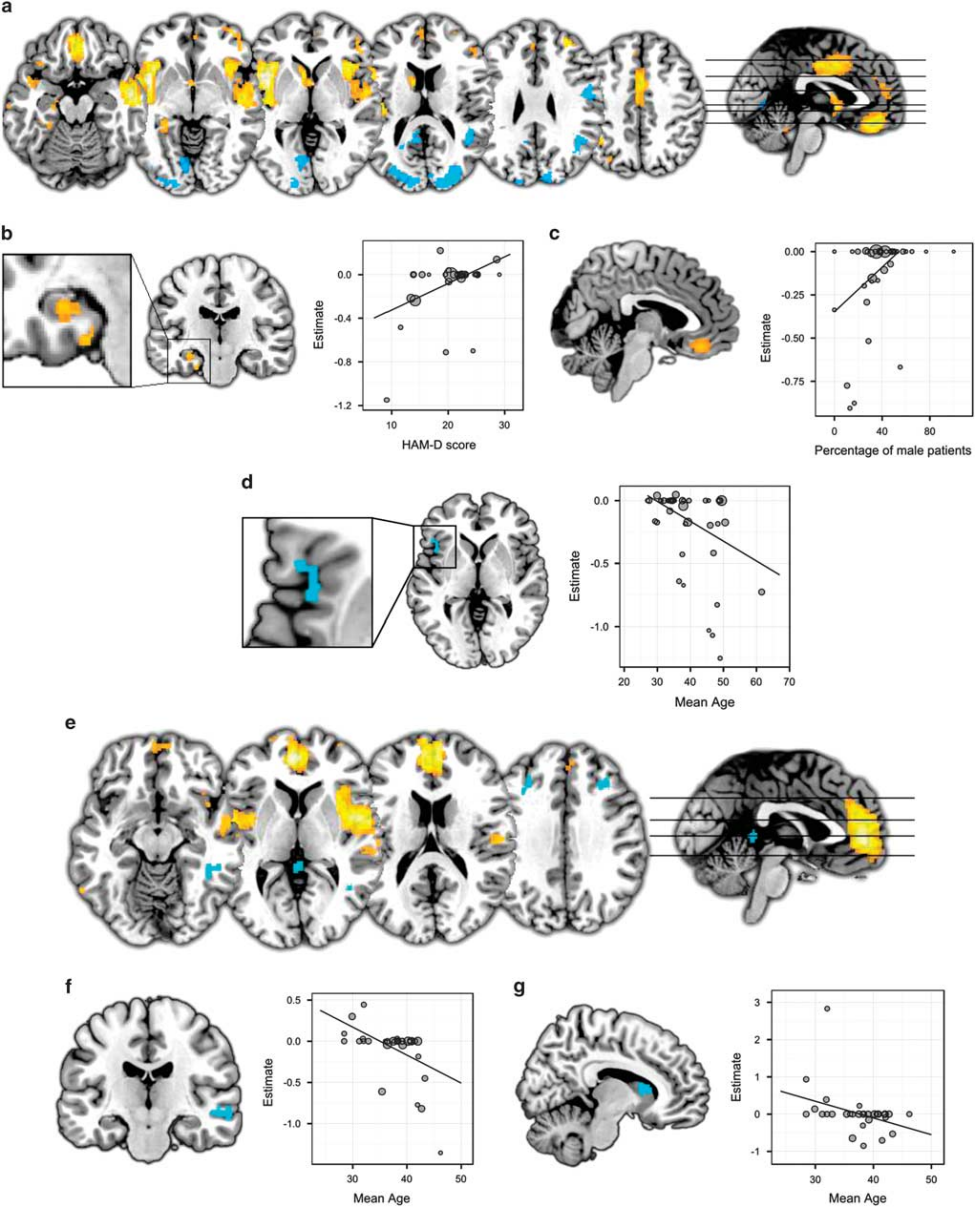
(**a**) Results of major depressive disorder (MDD) meta-analysis. (**b**) Results of meta-regression with depression severity in MDD. (**c**) Results of meta-regression with sex in MDD. (**d**) Results of meta-regression with patient age in MDD. (**e**) Results of bipolar disorder (BD) meta-analysis. (**f, g**) Results of meta-regressions with age in BD. Orange represents lower volume in patients relative to controls or positive relationships with regressors in meta-regressions, blue represents greater volume relative to controls or negative relationships with regressors. In meta-regression plots, point size represents study weights. All images are shown in neurological convention; left on the image corresponds to left in the brain. Effect sizes represent effect sizes at the peak of the cluster.

Grey-matter volume differences in MDD relative to healthy controls are shown in Table 1 and Figure 1a. Clusters that did not meet criteria for robustness are shown in Supplementary Table 3. The largest regions showing smaller grey-matter volume in MDD were observed bilaterally in two clusters comprising the insula, extending into the posterior part of the inferior frontal gyrus and the anterior superior temporal gyrus. The ventromedial prefrontal cortex showed lower volume in a large area, which was predominantly inferior to the anterior cingulate cortex. The posterior cingulate cortex and dorsal anterior cingulate cortex also exhibited lower volumes. Several lateral prefrontal regions showed smaller volumes, as did the left inferior parietal gyrus and the right fusiform gyrus. Regions of lower volume were also present in a number of subcortical and medial temporal regions, including the left caudate, left hippocampus and left parahippocampal gyrus.

Regions of greater volume relative to healthy controls were observed in the bilateral superior occipital gyrus, extending into the cuneus. Smaller clusters showing greater volume were found in the right angular gyrus and right postcentral gyrus.

There was no evidence of publication bias or detectable small-study effects in any cluster, as indicated by non-significant Egger’s tests of funnel plot asymmetry (all *P-*values >0.05). Details of brain regions where significant heterogeneity was measured are provided in Supplementary Table 4. Significant between-study heterogeneity was explored with meta-regression analyses. Results of these analyses indicated that studies with lower mean depression severity found smaller grey-matter volumes relative to controls in the left hippocampus (peak MNI=−30, −18, −16, *Z*=2.73, *P*<0.001, 40 voxels), studies with a smaller proportion of men found smaller grey-matter volume compared with controls in bilateral ventromedial prefrontal cortex (peak MNI=0, 38, −18, *Z*=2.17, *P*<0.001, 359 voxels) and studies with older patients found smaller volumes relative to controls in the left insula (peak MNI=−42, 16, −2, *Z*=−2.73, *P*<0.001, 49 voxels; Figures 1b–d). We found no association with antidepressant medication use or depression duration. When examining methodological variables, studies using higher field strength scanners showed smaller volumes relative to controls in the left superior temporal gyrus (peak MNI=−50, 0, −2, *Z*=−2.53, *P*<0.001, 40 voxels), while the opposite pattern was observed in the ventromedial prefrontal cortex (peak MNI=−2, 40, −18, *Z*=2.0, *P*<0.001, 187 voxels; Supplementary Figure S2).

#### Bipolar disorder vs healthy controls

Patients with BD differed from healthy controls in grey-matter volume in a number of regions (Table 2 and Figure 1e). Clusters that did not meet criteria for robustness are shown in Supplementary Table 5. The largest areas showing lower grey-matter volume in patients relative to controls were in the bilateral insula and superior temporal gyrus. Another large cluster where smaller volumes were observed was located in the medial prefrontal cortex, including the anterior cingulate cortex. We also found small clusters showing greater volume relative to controls in a number of areas, including a number of cerebellar regions, bilateral middle frontal gyrus, right middle and inferior temporal gyrus, and right middle occipital gyrus. Regions in which BD showed greater volume relative to controls included the inferior temporal gyrus and bilateral middle frontal gyrus, as well as cerebellar and occipital areas.

Egger’s test of funnel plot asymmetry did not identify any evidence of publication bias in any cluster (all *P-*values >0.05). A number of regions showed significant between-study heterogeneity (Supplementary Table 6). Meta-regression analyses revealed that smaller volumes relative to controls were associated with increasing age in the right middle temporal gyrus (Figure 1f, peak MNI=62, −26, −6, *Z*=−3.07, *P*<0.001, 186 voxels). Higher patient age was also associated with smaller volumes compared with controls in the right caudate (Figure 1g, peak MNI=8, 14, 12, *Z*=−2.60, *P*<0.001, 55 voxels). We found no significant associations with mood state, antipsychotic medication use, lithium use, sex or methodological variables.

#### MDD vs BD contrast

**Figure 2:**
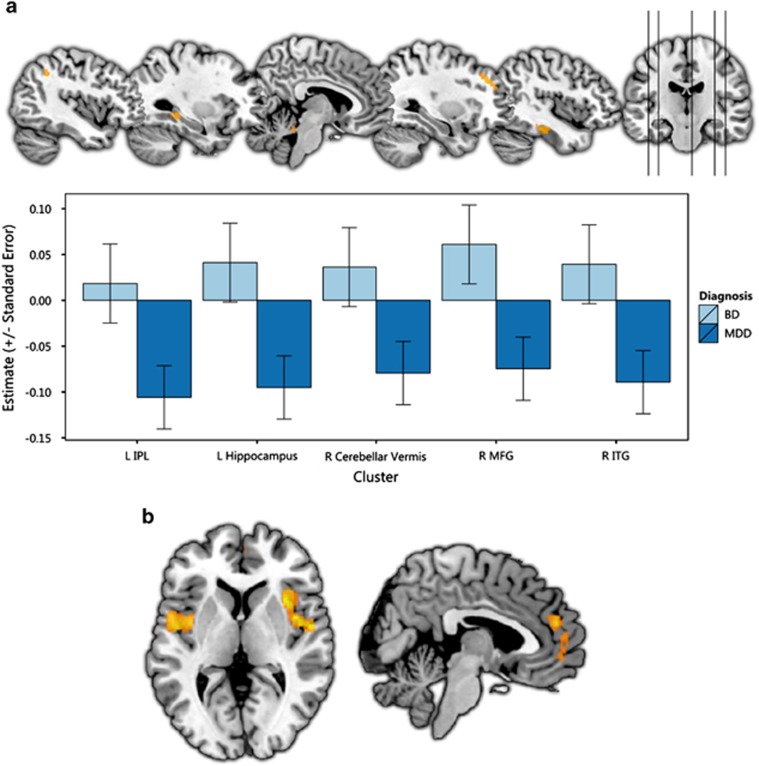
(**a**) Regions showing differences between major depression disorder (MDD) and bipolar disorder (BD). Orange clusters represent smaller grey-matter volume than controls, which is more substantial in MDD. (**b**) Results of the conjunction analysis showing regions with similar volumetric alterations in both conditions. Here, orange represents regions showing significantly lower volume in both conditions relative to controls. IPL, inferior parietal lobule; ITG, inferior temporal gyrus; L, left; MFG, middle frontal gyrus; R, right. Effect sizes represent effect sizes at the peak of the cluster.

MDD differed from BD with respect to grey-matter volume alterations in several regions (Table 3 and Figure 2a). The most substantial difference involved the right middle frontal gyrus, where smaller grey-matter volume relative to controls was specific to MDD. A similar pattern was found in the left hippocampus, right inferior temporal gyrus, left inferior parietal lobule and right cerebellar vermis. There were no regions in which the opposite pattern was observed.

#### Grey-matter volume alterations common to both disorders

Conjunction analysis indicated that several regions in the bilateral insula and in the dorsomedial and ventromedial prefrontal cortex, including the pre-genual anterior cingulate cortex, showed smaller volume compared with controls in both conditions (Figure 2b). No regions showed greater volume compared with controls in both conditions.

## Discussion

In this paper, we report findings from the largest meta-analysis conducted to date of VBM studies in MDD and BD. We compared results from these two conditions to identify common and distinct patterns of grey-matter volume alterations. We showed that the two conditions share similar patterns of lower volume in the bilateral insula and medial prefrontal cortex, suggesting that there may be an underlying pathological mechanism that is common to affective disorders. A number of regions, including the left hippocampus and right dorsolateral prefrontal cortex, differed between conditions, indicating that these disorders may be associated with spatially distinct patterns of pathophysiology.

Both conditions showed smaller grey-matter volumes relative to control groups in the medial prefrontal systems, including the anterior cingulate cortex. In MDD, this was predominantly located in the most ventral and dorsal regions of the medial prefrontal cortex, while in BD it was located in a large cluster anterior to the genu of the corpus callosum, although this difference in location was not statistically significant. The conjunction analysis indicated that the volumes of parts of the dorsomedial and ventromedial prefrontal cortex were robustly lower in both conditions, suggesting a consistent pattern across disorders. These regions have been strongly implicated in mood regulation, and the anterior cingulate cortex in particular has been shown to be crucial in the regulation of affective states,^27^ and has been a target of treatment with deep brain stimulation.^28^ Our results are consistent with theories of mood dysregulation in affective disorders that posit that dysfunction in regions such as the medial prefrontal cortex leads to aberrant mood states.^29^ Further work is necessary, however, to determine whether the structural differences here are responsible for the altered function of these networks.

We also found that bilateral insula volume was smaller in patents in both conditions. This region is involved in a range of functions, including affective processing and awareness of bodily states,^30, 31^ and atypical functioning of this region in affective disorders has been found in functional neuroimaging research.^32, 33^ Notably, the insula has also been found by multiple studies to predict treatment response in patients with depression.^34, 35^ Our finding of smaller insular volume in both unipolar and bipolar subjects suggests that structural abnormalities are present in the same areas in which altered function has also been identified. The insula has been implicated in interoceptive processing and general bodily awareness,^36^ and our results may indicate that structural changes are associated with altered interoceptive function in affective disorders;^37, 38^ this is a speculative interpretation, however, that requires direct testing.

Our comparison of the conditions revealed several areas of smaller grey-matter volume that were significantly greater in MDD than in BD, most prominently in the left parahippocampal gyrus and right dorsolateral prefrontal cortex, specifically the middle frontal gyrus. Smaller volumes of the hippocampus and parahippocampal gyrus have been well documented in MDD, but have been reported less often in BD.^12, 14, 39^ Investigators have suggested that this may be due to neuroprotective effects of lithium, which counteracts volume loss in BD.^40^ We did not find any significant heterogeneity in the hippocampus in BD, suggesting that there was no variation in effect sizes due to medication or other variables. It is important to note, however, that we cannot exclude the possibility that this may be due to reduced sensitivity of whole-brain VBM analyses in small regions such as the hippocampus.^41^ Additionally, it is unlikely that this difference between conditions can be explained by mood state in MDD given that our meta-regressions showed that lower depression severity in MDD was associated with smaller volumes in this region. Alternatively, this may reflect sparing of the hippocampus in BD due to protective factors in individuals predisposed to the disorder.^42^

The dorsolateral prefrontal cortex has been linked to emotion regulation,^43^ and the right dorsolateral prefrontal cortex specifically has been associated with attentional control during emotional tasks.^44^ Notably, repetitive transcranial magnetic stimulation to this region has been reported to improve symptoms in treatment-resistant depression,^45^ but results have been less convincing in bipolar depression.^46^ Our findings add to this literature by suggesting that volumetric alterations in this region are specific to MDD, indicating that a differential pattern of prefrontal grey-matter volume may potentially differentiate these two disorders. It is important to mention that functional alterations have been identified in the right dorsolateral prefrontal cortex in BD.^47^ The relationship between functional and structural alterations in these conditions remains unclear; and further research is essential to understand potential functional and/or structural disease-specific alterations within affective disorders in the dorsolateral prefrontal cortex.

Our analyses within each condition also revealed a number of areas of grey-matter changes that did not differ significantly in magnitude between disorders but that were not reliably smaller in both disorders relative to controls. One notable difference in MDD compared with controls involved the bilateral occipital cortex, including primary visual and extrastriate cortices, where patients showed a large area of greater volume relative to controls. Although a number of studies have highlighted the possibility of neurochemical^48^ and functional^49, 50^ changes in these regions, we believe this is the first study to identify robust volumetric changes in these locations. Given our efforts to ensure that our results were reliable and robust, it is unlikely that this is simply a false positive produced by the meta-analytic method, although we cannot exclude the possibility that methodological issues in the original studies may have caused spurious results. For example, it is possible this could be an artefact caused by correction for intracranial or total grey-matter volume combined with substantially lower grey-matter volume in other areas, although this is a speculative interpretation and would require confirmation. The potential role of occipital regions has rarely been investigated in major depression, and further research is required to understand whether these results are robust or whether they are a result of the method used.

We found a number of regions that showed significant between-study heterogeneity, and we explored these using meta-regressions. In MDD, studies with less severely depressed patients showed smaller grey-matter volume in the hippocampus than did investigations with more severe patients. This may seem contradictory given that previous studies have showed the opposite pattern.^51, 52^ It is possible that it may be explained by the use of medication. Treatment with selective serotonin reuptake inhibitors is known to increase hippocampal volume,^53, 54^ and given this it is possible that more severely depressed patients had received more extensive pharmacotherapy in the past, leading to amelioration of pre-existing grey-matter volumetric abnormalities, although we were unable to test this here due to historical treatment data being unavailable in the original studies. However, it is important to note that we only had access to information regarding current depression severity, and it is possible that lifetime depression severity, or chronic and treatment-resistant symptom profiles, may be associated with different neuroanatomical profiles.

Our meta-regression analyses showed effects of demographic variables in both conditions. In MDD, volume of the prefrontal cortex was smaller in studies with fewer male patients. Anatomical differences between sexes have been reported previously in depression,^55^ although it is unclear what drives these differences. In addition, we found smaller left insula volumes in studies of MDD with older patients; in contrast, in BD studies with older patients, we found smaller volumes in the right middle temporal gyrus. Thus, there may be a different biological trajectory in affective disorders in relation to these regions, although meta-regressions should be interpreted with caution as they do not directly test relations within samples.

This meta-analysis improves on previous studies in several ways. First, the novel meta-analytic method used here allowed us to identify common and distinct areas of grey-matter volume alterations in affective disorders. Given the paucity of reports comparing affective conditions directly, this approach enabled us to identify volumetric aspects of common neuropathological mechanisms, and potentially distinctive biomarkers. Second, we were able to include a larger number of studies due to the rapid growth of the field and our access to as-yet unpublished data sets. We are therefore able to provide the most conclusive picture of volumetric changes currently available. Third, we included a number of original statistical maps in our analyses. This substantially improves the sensitivity and specificity of the analysis, especially in cases in which individual studies have small samples.^19^ Finally, the thorough and detailed approach used in this work ensured that findings were robust and that heterogeneity was comprehensively explored. We found no evidence of publication bias or small-study effects; nevertheless, it is important to note that, given the small sample sizes of the majority of the studies included in the analyses, we cannot exclude the possibility of small-study bias.

Despite these strengths, we should also note several limitations of this meta-analysis. First, we cannot determine causality from these results due to the fact that all the included studies were cross-sectional group comparisons, and it is not clear whether these alterations are part of the pathogenesis of these disorders or a consequence of the illness. It should be noted however that our meta-regressions did not detect any effect of illness duration, providing some evidence against the latter explanation. Second, the effect size comparisons may not provide as accurate a picture of group differences as studies directly comparing the two conditions. To date, there have been very few VBM studies directly comparing affective conditions^15, 16^ making it difficult to draw firm conclusions concerning potential similarities and differences between disorders. Hence, at present, the approach used in this meta-analysis, with the limitation of indirectly comparing studies’ effect sizes, offers the most viable option to reach conclusions generalisable beyond individual studies. Additionally, given the inherent robustness of the meta-analytic method, our results should provide a summary of the most reliable differences between these disorders.

Third, the samples used in the studies differed between disorders with respect to treatment status (for example, different types of pharmacotherapy). Given that psychotropic medications can have effects on brain structure,^53^ it is difficult to be certain that results are not entirely independent from medication status. As a related point, the samples also differed in mood state and illness duration. We found no evidence for effects of these variables in meta-regressions within disorders, suggesting that this is not likely to be a major concern. However, effects of mood state are particularly difficult to rule out, given that a number of studies included samples of mixed mood states and several did not provide information on mood state. Consequently, it is not possible to comment with certainty on the effect of mood state on our results. Furthermore, we cannot exclude the possibility that undiagnosed cases of BD presented as unipolar depression in the original studies,^6^ and it is not possible to rule out the influence of comorbidities such as anxiety disorders on the results as these were not always well described in the original reports. Another concern is that many of the retrieved studies included more controls than patients. Although these unbalanced studies may have theoretically biased results,^56^ it is not clear from the existing literature that this is likely to affect our findings and conclusions.

Finally, we cannot be certain that these regions of common grey-matter volume alterations are exclusive to affective disorders. A recent meta-analysis by Goodkind *et al.*^57^ found that some of these areas, such as the insula and dorsomedial prefrontal cortex, are lower in volume across a range of psychiatric conditions including affective, anxiety and psychotic disorders. This suggests that morphometric grey-matter changes in these regions are not specifically pathognomonic to affective disorders, or are even a proxy for underlying common disease processes or for risk factors such as life stressors or effects of hormonal or inflammatory changes. Nevertheless, the locations identified by Goodkind *et al.* differ from those reported in this meta-analysis in their location and size. For example, the authors demonstrated that the anterior left insula extending to the left inferior frontal gyrus was affected across disorders. In our work, a more posterior portion of the left insula was shown to be affected in both MDD and BD, which has functional relevance given the anterior-posterior division in insula function, with the posterior region being specifically involved in interoception and bodily awareness.^36^

In conclusion, we have shown that MDD and BD show a common pattern of lower grey-matter volume which predominantly includes the medial prefrontal and insular cortices. In addition, the two conditions also show distinct patterns of volume alterations in a number of other regions, most predominantly the right dorsolateral prefrontal cortex and left hippocampus, which are specific to MDD. There is significant heterogeneity within these results, but this could be partially explained by clinical and demographic differences in clinical samples. These findings suggest targets for neuroanatomical diagnostic biomarkers, but also indicate that affective disorders are more morphologically similar than they are different.

## Figures and Tables

**Table 1:** Clusters showing differences between MDD and controls that met our criteria for robustness

*Peak MNI coordinate*	*Z*	*P*	*Voxels*	*Brodmann areas*	*Regions*
*MDD<Healthy controls*					
−42, 8, −2	4.05	<0.001	3258	22, 38, 48	Left insula, inferior frontal gyrus, temporal pole, superior temporal gyrus
54, −8, −14	4.00	<0.001	1912	21, 22, 48	Right superior temporal gyrus, insula, inferior frontal gyrus
−2, 40, −18	3.40	<0.001	908	11	Left gyrus rectus, left medial orbitofrontal cortex, anterior cingulate cortex
0, 4, 42	3.34	<0.001	729	23, 24	Left middle cingulate cortex
−10, 16, 6	3.53	<0.001	320	25	Left caudate nucleus
44, 48, −8	3.12	<0.001	282	46, 47	Right middle frontal gyrus, orbital part
32, 42, 30	3.10	<0.001	144	46	Right middle frontal gyrus
−28, −38, −4	2.88	0.001	104	37	Left hippocampus, parahippocampal gyrus
−40, −54, 46	3.21	<0.001	93	40	Left inferior lobule
44, −24, −24	2.78	0.001	92	20	Right fusiform gyrus
4, 48, 22	2.80	0.001	53	32	Right anterior cingulate cortex
−20, −18, −18	2.73	0.001	41	35	Left hippocampus, parahippocampal gyrus
−4, 36, 40	2.67	0.001	36	32	Left superior medial frontal gyrus
					
*MDD>Healthy controls*					
26, −90, 14	−1.81	~0	731	17, 18	Right superior occipital gyrus, cuneus, middle occipital gyrus
−10, −96, 12	−1.03	<0.001	733	17, 18	Left superior occipital gyrus
44, −50, 26	−1.33	<0.001	457	39	Right angular gyrus, middle temporal gyrus
52, −4, 26	−1.25	<0.001	161	4	Right postcentral gyrus

Abbreviation: MDD, major depressive disorder.

**Table 2:** Clusters showing differences between BD and controls that met our criteria for robustness

*Peak MNI coordinate*	*Z*	*P*	*Voxels*	*Brodmann areas*	*Regions*
*BD<Healthy controls*					
−4, 50, 4	4.04	<0.001	2210	10, 32	Bilateral anterior cingulate cortex, superior and ventral medial prefrontal cortex
54, 2, 0	3.95	<0.001	1898	21, 22, 38, 48	Right temporal pole, superior temporal gyrus, right insula
−48, −2, 0	3.06	<0.001	436	48	Left superior temporal gyrus, left insula, left rolandic operculum
					
*BD>Healthy controls*					
40, −44, −12	−1.56	<0.001	158	20, 21, 37	Right inferior temporal gyrus, middle temporal gyrus
24, −36, −38	−1.59	<0.001	127	—	Middle cerebellar peduncles
34, 26, 36	−1.73	<0.001	84	46	Right middle frontal gyrus
−32, 22, 38	−1.41	0.001	71	46, 9	Left middle frontal gyrus
2, −38, 6	−1.54	0.001	54	—	Cerebellar vermis
38, −78, 8	−1.35	0.001	15	19	Right middle occipital gyrus

Abbreviation: BD, bipolar disorder.

**Table 3:** Clusters showing similar and different grey-matter changes in MDD and BD

*Peak MNI coordinate*	*Z*	*P*	*Voxels*	*Brodmann areas*	*Regions*
*MDD<BD*					
34, 30, 40	−2.46	<0.001	102	9, 46	Right middle frontal gyrus
−26, −38, −2	−2.47	<0.001	74	37	Left hippocampus, parahippocampal gyrus
42, −26, −22	−2.33	<0.001	72	20	Right inferior temporal gyrus, fusiform gyrus
−40, −52, 44	−2.25	<0.001	31	40	Left inferior parietal lobule
4, −42, −22	−2.10	<0.001	14	—	Right cerebellar vermis
					
*Reductions in both MDD and BD*					
52, −4, 2	4.97	<0.001	753	48	Right superior temporal gyrus, insula
−42, 0, −2	4.69	<0.001	377	38, 48	Left insula, superior temporal gyrus
−4, 54, 18	4.28	0.001	115	10, 32	Left superior medial frontal gyrus, anterior cingulate cortex
4, 48, 22	4.20	0.001	50	32	Right anterior cingulate cortex

Abbreviations: BD, bipolar disorder; MDD, major depression disorder.
