# Supplementary material for: Common and distinct patterns of grey-matter volume alteration in major depression and bipolar disorder: evidence from voxel-based meta-analysis
Source: Mol Psychiatry. 2016 May 24;22(10):1455–63. doi: 10.1038/mp.2016.72 (PMC5622121; doi:10.1038/mp.2016.72)
Supplement: Supplementary Information [file mp201672x1.docx]

**Supplementary methods**

*Meta-analysis*

All analyses were conducted using the grey matter template included in AES-SDM using a voxel size of 1mm^3^. We used a significance threshold of p < .005 with a peak Z value of >1 and cluster size of >10 voxels for all main analyses. We used a more conservative threshold of p < .0005 for meta-regressions as these are more susceptible to false positives, particularly when conducted on a small number of studies^1^. These thresholds have been found to provide the optimal balance of sensitivity and specificity in previous research^2^. As we had a large number of studies in each diagnostic group, we used a less conservative threshold of p < .001 for the comparison between MDD and BD. Regions where results were significant in both conditions were submitted to a conjunction analysis to identify regions that were robustly affected in both conditions, accounting for error in the estimation of p-values within individual meta-analyses^3^.To protect against false positives in the conjunction analysis,the threshold was set at p < .005 for this analysis, without the correction described by Radua and others^3^ in order to test against the conjunction null hypothesis (i.e. no significant difference in one or fewer disorders) rather than the global null (i.e. no significant difference in any disorder).

**Supplementary results**

*Results of heterogeneity analysis in regions where there was significant heterogeneity but no main disorder effect – Bipolar Disorder*

We found that studies with euthymic samples found greater volume reductions in the right caudate than those in other mood states (peak MNI = -4, 10, 4, *Z* = 2.21, *p*< .001, 120 voxels, Figure 2A). Although our overall analysis showed no differences between patients and controls here, we performed a subgroup analysis using only studies with euthymic patients and found a cluster of significant volume reductions in the caudate (peak MNI = -6, 14, 0, *Z* = -3.61, *p*< .001, 804 voxels), suggesting that this may be specific to the euthymic state.

Meta-regression analysis also revealed that the percentage of patients taking antipsychotic medication at the time of scanning was positively associated with effect size in the left fusiform gyrus and parahippocampal gyrus (peak MNI = -30, -28, -28, *Z* = --2.20, *p*< .001, 134 voxels), indicating that studies with higher numbers of patients receiving antipsychotic treatment found greater decreases in grey matter volume in this region (Figure 2B). Again, this region was not present in our main analysis but was present when we conducted a subgroup analysis using only studies with >50% of patients taking antipsychotic medication (peak MNI = -34, -28, -32, *Z =* 2.01, *p* = 0.001, 95 voxels).Meta-regression analyses with methodological variables showed that studies with higher strength scanners showed smaller volumes relative to controls in the right caudate (peak MNI = 10, 6, 6, *Z =* 3.19,  *p*< .001, 597 voxels) and the right superior temporal gyrus (peak MNI = 62, -30, 2, *Z* = 2.57, *p*< .001, 269 voxels). Meta-regression analysis with spatial smoothing level revealed a negative relationship between smoothing level and effect size in the left inferior frontal gyrus (peak MNI = -44, 16, 4, *Z* = 4.14, *p*< .001, 101 voxels).

All other meta-regression results were non-significant, or did not overlap with regions of heterogeneity.

| **Supplementary tables** | | | | | | | | | | | | | | |
| --- | --- | --- | --- | --- | --- | --- | --- | --- | --- | --- | --- | --- | --- | --- |
|  | **Major Depression Patients** | | | | | | |  | **Healthy Controls** | | |  | **Methods** | |
| **Study** | ***n*** | **Mean age** | **Sex (M/F)** | **Antidepressant %** | **Duration of illness (y)** | **State** | **HAMD-17** |  | ***n*** | **Mean age** | **Sex (M/F)** |  | **Field strength (Tesla)** | **Smoothing**  **(mm)** |
| Abe (2010)^4^ | 21 | 48.1 | 11, 10 | 90 | 6 | 7 Depressed, 2 partially remitted, 12 fully remitted | 9.2 |  | 42 | 48 | 22, 20 |  | 1.5 | 6 |
| Amico (2011)^5^ | 33 | 32 | 19, 14 | 82 | 3.4 | Depressed | 18.6 |  | 64 | 30.4 | 36, 28 |  | 1.5 | 8 |
| Arnone (2012a)^6^ | 39 | 36.3 | 12, 27 | 0 | 14.3 | Depressed | 21.8 |  | 66 | 32.1 | 20, 46 |  | 1.5 | 8 |
| Arnone (2012b)^6^ | 25 | 34.5 | 5, 20 | 0 | 9.4 | Remitted | 0.3 |  | 66 | 32.1 | 20, 46 |  | 1.5 | 8 |
| Bergouignan (2009)^7^ | 20 | 33.2 | 3, 17 | 100 | 8.5 | Depressed | 23.1 |  | 21 | 28.2 | 7, 14 |  | 1.5 | 8 |
| Chaney (2014)^8^ | 37 | - | - | - | - | Depressed | - |  | 46 | - | - |  | 3 | 10 |
| Cheng (2010)^9^ | 68 | 29.9 | 21, 47 | 0 | 11 | Depressed | 22.3 |  | 68 | 30.5 | 21, 47 |  | 1.5 | 10 |
| de Azevedo-Marques Périco (2011)^10^ | 20 | 29.9 | 5, 15 | 55 | 0.7 | - | 19.6 |  | 94 | 30.2 | 41, 53 |  | 3 | 8 |
| Grieve (2013)^11^ | 102 | 33.8 | 48, 54 | 0 | 11.3 | Depressed | 21 |  | 34 | 31.5 | 18, 16 |  | 3 | 8 |
| Guo (2014)^12^ | 44 | 27.5 | 22, 22 | 0 | 1.6 | Depressed | 25.2 |  | 44 | 29.4 | 20, 24 |  | 1.5 | 10 |
| Inkster (2011a)^13^ | 49 | 47.6 | 16, 33 | - | 14.1 | - | - |  | 183 | 48 | 73, 110 |  | 1.5 | 10 |
| Inkster (2011b)^13^ | 96 | 50.4 | 35, 61 | - | 14.5 | - | - |  | 183 | 48 | 73, 110 |  | 3 | 6 |
| Jia (2010a)^14^ | 36 | 34.7 | 20, 16 | 0 | 1.8 | Depressed | 22.3 |  | 52 | 37.1 | 24, 28 |  | 3 | 6 |
| Jia (2010b)^14^ | 16 | 34.2 | 5, 11 | 0 | 6.7 | Depressed | 24.6 |  | 52 | 37.1 | 24, 28 |  | 1.5 | 8 |
| Kim (2008)^15^ | 22 | 38.5 | 0, 22 | 45 | 17.4 | Depressed | - |  | 25 | 35.3 | 0, 25 |  | 1.5 | 8 |
| Klauser (2015)^16^ | 56 | 34 | 16, 40 | - | 10.3 | 29 depressed, 27 remitted | - |  | 33 | 34.7 | 12, 21 |  | 1.5 | 8 |
| Kong (2014)^17^ | 28 | 34.4 | 11, 17 | 0 | 2.11 | Depressed | 21.7 |  | 28 | 32.1 | 14, 14 |  | 1.5 | 8 |
| Kroes (2011)^18^ | 29 | 33.4 | 8, 21 | 76 | - | Depressed | - |  | 29 | 32.5 | 13, 16 |  | 3 | 3 |
| Lai (2010)^19^ | 16 | 37.9 | 5, 11 | 0 | 0.4 | Depressed | 29.1 |  | 15 | 34.3 | 4, 11 |  | 3 | 3 |
| Lai and Wu (2014)^20^ | 38 | 36.6 | 18, 20 | 0 | 0.4 | Depressed | 22.3 |  | 27 | 38.3 | 12, 15 |  | 1.5 | 8 |
| Lee (2011)^21^ | 47 | 46 | 5, 42 | 62 | 3.9 | Depressed | 20.1 |  | 51 | 45.7 | 5, 46 |  | 1.5 | 12 |
| Leung (2009)^22^ | 17 | 45.5 | 0, 17 | - | 7 | Depressed | - |  | 17 | 45.8 | 0, 17 |  | 1.5 | 8 |
| Ma (2012a)^23^ | 18 | 27.4 | 11, 7 | - | 3 | Depressed | 23.9 |  | 17 | 24.2 | 10, 7 |  | 1.5 | 8 |
| Ma (2012b)^23^ | 17 | 26.7 | 10, 7 | 0 | 0.22 | Depressed | 25.6 |  | 17 | 24.2 | 10, 7 |  | 1.5 | NA |
| MacGregor Legge (2015)^24^† | 67 | 50.5 | 28, 39 | 67 | 21.8 | Depressed and remitted | - |  | 67 | 26 | 23, 44 |  | 1.5 | 8 |
| Machino (2014)^25^ | 29 | 39.6 | 16, 13 | 97 | 4.4 | Depressed | 13.9 |  | 29 | 38.7 | 16, 13 |  | 1.5 | 8 |
| Modinos (2014)^26^ | 23 | 44.6 | 3, 20 | - | - | - | - |  | 46 | 25.3 | 32, 14 |  | 1.5 | 8 |
| Nakano (2014)^27^ | 36 | 49 | 14, 22 | - | 5.6 | Depressed & remitted | 15.4 |  | 54 | 45.4 | 27, 27 |  | 3 | 8 |
| Peng (2011)^28^ | 22 | 46.7 | 8, 14 | 23 | 0.7 | Depressed | 19.5 |  | 30 | 45.9 | 11, 19 |  | 3 | 8 |
| Peng (2014a)^29^ | 18 | 31.1 | 6, 12 | - | 23.5 | Depressed | 24.1 |  | 28 | 28.6 | 15,13 |  | 3 | 12 |
| Peng (2014b)^29^ | 20 | 27.8 | 7, 13 | - | 21.5 | Depressed | 25.6 |  | 28 | 28.6 | 15,13 |  | 1.5 | 8 |
| Redlich (2014)^30^ | 58 | 37.6 | 22, 36 | - | 11 | Depressed | 22.4 |  | 58 | 37.7 | 21, 37 |  | 3 | 8 |
| Rodríguez-Cano (2014)^31^ | 32 | 48.7 | 12, 20 | 88 | 11 | Depressed | 21.7 |  | 64 | 46 | 26, 38 |  | 1.5 | 4 |
| Salvadore (2011a)^32^ | 58 | 38.8 | 21, 37 | 0 | 18.4 | Depressed | 20.8 |  | 107 | 36.2 | 47, 60 |  | 3 | 11 |
| Salvadore (2011b)^32^ | 27 | 40.2 | 6, 21 | 0 | 15.1 | Remitted | 0 |  | 107 | 36.2 | 47, 60 |  | 3 | 11 |
| Scheuerecker (2010)^33^ | 13 | 37.9 | 10, 3 | 0 | 4.4 | Depressed | 16.6 |  | 15 | 35.5 | 10, 5 |  | 3 | 8 |
| Serra-Blasco (2013a)^34^ | 22 | 44 | 7, 15 | - | 0.5 | Depressed | 16.6 |  | 32 | 46 | 9, 23 |  | 3 | 8 |
| Serra-Blasco (2013b)^34^ | 22 | 48 | 2, 20 | - | 17.9 | Remitted | 4 |  | 32 | 46 | 9, 23 |  | 3 | 8 |
| Serra-Blasco (2013c)^34^ | 22 | 49 | 4, 18 | - | 22.7 | Depressed | 21 |  | 32 | 46 | 9, 23 |  | 3 | 8 |
| Shah (1998a)^35^ | 20 | 47.7 | 13, 7 | 45 | 1.6 | Remitted | 2.6 |  | 20 | 49.3 | 13, 7 |  | 1 | 12 |
| Shah (1998b)^35^ | 20 | 48.9 | 13, 7 | 100 | 5.5 | Depressed | 20.6 |  | 20 | 49.3 | 13, 7 |  | 1 | 12 |
| Soriano-Mas (2011)^36^ | 70 | 61.6 | 29, 41 | 71 | 10.6 | Depressed | 28.6 |  | 40 | 59.2 | 17, 23 |  | 1.5 | 12 |
| Sprengelmeyer (2011)^37^ | 17 | 45.6 | 8, 2 | 100 | - | Depressed | 23.2 |  | 21 | 42 | 9, 12 |  | 1.5 | 8 |
| Stratmann (2014)^38^ | 132 | 37.9 | 56, 76 | 94 | 7.8 | Depressed | 20.5 |  | 132 | 37.8 | 58, 74 |  | 3 | 8 |
| Treadway (2009)^39^ | 19 | 35.2 | 9, 10 | 0 | 12.9 | Depressed | 21.5 |  | 19 | 30.3 | 9, 10 |  | 3 | 12 |
| Wagner (2011)^40^ | 30 | 0 | 5, 25 | - | 6 | Depressed | 20.1 |  | 30 | 0 | 5, 25 |  | 1.5 | 12 |
| Wang (2012)^41^ | 18 | 34 | 9, 9 | 0 | 0.4 | Depressed | 25 |  | 18 | 35 | 9, 9 |  | 3 | 6 |
| Wang (2014)^42^ | 13 | 30.9 | 0, 13 | - | - | Depressed | - |  | 10 | 29.8 | 0, 10 |  | 3 | 4 |
| Yoshikawa (2006)^43^ | 11 | 48.5 | 0, 11 | 0 | - | Depressed | - |  | 29 | 48.6 | 0, 29 |  | 1.5 | 8 |
| Zou (2010)^44^ | 23 | 31.1 | 10, 13 | 0 | 0.6 | Depressed | 24.4 |  | 23 | 36.6 | 10, 13 |  | 3 | 10 |
| **Supplementary Table 1. Characteristics of major depression studies included in the meta-analysis.** M = male, F = female, y = years, HAMD-17 = Hamilton Depression Rating Scale 17-item, † = unpublished voxel-based morphometry data from the sample reported in this study. | | | | | | | | | | | |  |  |  |

|  | **Bipolar Disorder Patients** | | | | | | | | | |  | **Healthy Controls** | | |  | **Methods** | |
| --- | --- | --- | --- | --- | --- | --- | --- | --- | --- | --- | --- | --- | --- | --- | --- | --- | --- |
| **Study** | ***N*** | **Age** | **Sex (M,F)** | **Lithium (%)** | **Antipsychotic medication (%)** | **Duration of illness**  **(y)** | **Subtype** | **State** | **HAMD-17** | **YMRS** |  | ***N*** | **Age** | **Sex (M/F)** |  | **Field strength**  **(Tesla)** | **Smoothing**  **(mm)** |
| Adler (2005)^45^ | 32 | 31.2 | 19, 13 | - | - | 8.7 | BD-I | 5 manic,  2 depressed, 25 euthymic | - | - |  | 27 | 30.5 | 12, 15 |  | 3 | 12 |
| Almeida (2009)^46^ | 27 | **31.9** | 10, 17 | - | 59.3 | 11.1 | BD-I | 17 euthymic, 10 depressed | - | - |  | 28 | 30.8 | 13, 15 |  | 3 | 12 |
| Ambrosi (2013)^47^ | 20 | 42 | 5, 15 | 35 | 50.0 | 12.6 | BD-II | Euthymic | - | - |  | 21 | 34.6 | 6, 15 |  | 1.5 | 8 |
| Brown (2011)^48^ | 15 | 46.2 | 7, 8 | - | 20.0 | 19.1 | BD-I | Depressed | 7.9 | - |  | 21 | 45 | 10, 11 |  | 1.5 | 8 |
| Bruno (2004)^49^ | 39 | 39.1 | 13, 26 | 59 | 23.1 | 13.2 | 28 BD-I, 11 BD-II | - | - | - |  | 35 | 34.8 | 10, 25 |  | 1.5 | 8 |
| Chen (2007)^50^ | 24 | 38.2 | 6, 18 | 50 | 0.0 | 14.2 | BD-I | - | - | - |  | 25 | 38.4 | 7, 18 |  | 1.5 | 12 |
| Chen (2012)^51^ | 18 | 32 | 18, 0 | 83 | 16.7 | 4.2 | - | Manic | 3.2 | 24.8 |  | 27 | 31.3 | 27, 0 |  | 1.5 | 8 |
| Cui (2011)^52^ | 24 | 28.4 | 15, 9 | - | - | 6.1 | BD-I | Manic | - | 25.9 |  | 23 | 24.8 | 16, 7 |  | 3 | 6 |
| de Azevedo-Marques Périco (2011)^10^ | 26 | 29.9 | 5, 15 | 23.1 | 42.0 | 0.5 | BD-I | - | 7.5 | 7.4 |  | 94 | 30.2 | 41, 53 |  | 2 | 8 |
| Doris (2004)^53^ | 11 | 40.5 | 6, 5 | - | 45.5 | 16.2 | BD-I | Euthymic | 8.3 | - |  | 16 | 39.1 | 7, 9 |  | 3 | 8 |
| Eker (2014)^54^ | 28 | 36.4 | 16, 12 | 23 | 17.0 | 16.3 | BD-I | Euthymic | 2.3 | 1 |  | 30 | 34.7 | 10, 20 |  | 1.5 | 8 |
| Emsell (2013)^55^ | 60 | 42 | 31, 29 | 77 | 56.7 | 13 | BD-I | Euthymic | - | - |  | 60 | 42 | 31, 29 |  | 1.5 | 8 |
| Ha (2009a)^56^ | 23 | 35.2 | 8, 15 | 30 | 34.8 | 10.5 | BD-II | 7 depressed, 16 euthymic | 13.1 | - |  | 23 | 36 | 8,15 |  | 1.5 | 8 |
| Ha (2009b)^56^ | 23 | 35.6 | 8,15 | 35 | 43.5 | 10.4 | BD-I | 4 depressed, 19 euthymic | 8.8 | - |  | 23 | 36 | 8,15 |  | 1.5 | 5 |
| Haldane (2008)^57^ | 44 | 42.7 | 20, 24 | - | 50.0 | 16.3 | BD-I | Euthymic | 4 | 1.2 |  | 44 | 43.1 | 20, 24 |  | 1.5 | 8 |
| Kempton (Unpublished)^58^ | 26 | 42.1 | 9, 17 | 35 | 15.0 | 16.1 | 24 BD-I, 2 BD-II | Euthymic | - | - |  | 23 | 41.2 | 7, 16 |  | 3 | 8 |
| Li (2011)^59^ | 24 | 28.4 | 15, 9 | 83 | - | 6 | BD-I | 7 depressed, 17 manic | 20 | 25.9 |  | 36 | 26.6 | 21, 15 |  | 1.5 | 12 |
| Lochhead (2004)^60^ | 11 | 38.2 | 6, 5 | - | - | 13.9 | 7 BD-I, 4 BD-II | 11 depressed | 18 | - |  | 31 | 36 | 16, 15 |  | 1.5 | 8 |
| Lyoo (2004)^61^ | 39 | 38.3 | 16, 23 | 26 | - | 19.7 | BD-I | 22 depressed, 17 manic | - | - |  | 43 | 35.7 | 19, 24 |  | 1.5 | 8 |
| McDonald (2005)^62^ | 37 | 40.7 | 15, 22 | 59 | 24.3 | 17.8 | BD-I | - | - | - |  | 52 | 39.3 | 24, 28 |  | 1.5 | 8 |
| McIntosh (2004a)^63^ | 26 | 40.5 | 14, 12 | - | - | - | BD-I | - | - | - |  | 54 | 35.3 | 23, 26 |  | 1.5 | 8 |
| McIntosh (2004b)^63^ | 19 | 39.7 | 7, 12 | - | - | - | BD-I | - | - | - |  | 54 | 35.3 | 23, 26 |  | 1.5 | 8 |
| Molina (2011)^64^ | 19 | 38.3 | 12, 7 | 84 | 5.3 | 12 | BD-I | Euthymic | - | - |  | 24 | 34.6 | 16, 8 |  | 1.5 | 12 |
| Narita (2011a)^65^ | 17 | 41.4 | 9, 8 | 65 | 11.8 | 6.2 | BD-II | 6 euthymic, 9 depressed, 2 manic | - | - |  | 84 | 41.1 | 48, 36 |  | 1.5 | 12 |
| Narita (2011b)^65^ | 14 | 40.2 | 8, 6 | 76 | 21.4 | 8.6 | BD-II | 2 euthymic, 10 depressed, 2 manic | - | - |  | 84 | 41.1 | 48, 36 |  | 3 | 8 |
| Nugent (2006a)^66^ | 16 | 37 | 5, 11 | 0 | 0.0 | 17 | - | Depressed | - | - |  | 65 | 38 | 19, 46 |  | 3 | 8 |
| Nugent (2006b)^66^ | 20 | 41 | 5, 15 | 31 | 2.8 | 23 | - | Depressed | - | - |  | 65 | 38 | 19, 46 |  | 1.5 | 8 |
| Redlich (2014)^30^ | 58 | 37.5 | 21, 37 | 26 | 64.0 | 14.2 | BD-I | Depressed | 21 | 3.0 |  | 58 | 37.7 | 21, 37 |  | 3 | 8 |
| Rocha-Rego (2013a)^67^ | 26 | 41.5 | 12, 14 | 38 | 30.8 | 15.8 | BD-I | Euthymic | 6.6 | 1.8 |  | 26 | 41.3 | 12, 14 |  | 1.5 | 8 |
| Rocha-Rego (2013b)^67^ | 14 | 37.6 | 6. 8 | - | 0.0 | 18.8 | BD-I | Euthymic | 4.2 | 1.7 |  | 14 | 37.4 | 6, 8 |  | 1.5 | 8 |
| Scherk (2008)^68^ | 35 | 43.3 | 18, 17 | 34 | 51.4 | 14.4 | BD-I | Euthymic | 2.5 | 2.6 |  | 32 | 33.7 | 12, 20 |  | 1.5 | 8 |
| Shepherd (2014)^69^ | 30 | 39.1 | 12, 18 | - | - | 13.5 | BD-I | - | - | 6.7 |  | 34 | 32.6 | 16, 18 |  | 3 | 8 |
| Stanfield (2009)^70^ | 66 | 36.4 | 30, 36 | - | 47.0 | 15.4 | BD-I | Euthymic | - | - |  | 66 | 39 | 31, 35 |  | 1.5 | 12 |
| Tang (2014)^71^ | 27 | 32 | 10, 17 | 33 | - | 4.2 | - | Depressed | 19.7 | 1 |  | 27 | 32.6 | 11, 16 |  | 3 | 8 |
| Yatham (2007)^72^ | 15 | 36 | 6, 9 | - | 0.0 | 3.9 | BD-I | Manic | - | 27 |  | 15 | 36 | 6, 9 |  | 1.5 | 8 |
| Yüksel (2012)^73^ | 27 | 32.9 | 17, 10 | 48 | 0.0 | - | BD-I | 18 manic, 5 mixed, 4 euthymic | 7.2 | 22.8 |  | 43 | 36.4 | - |  | 3 | 12 |
| **Supplementary Table 2. Characteristics of bipolar disorder studies included in the meta-analysis.** M = male, F = female, y = years, HAMD-17 = Hamilton Depression Rating Scale 17-item, BD-I = bipolar disorder type I, BD-II = bipolar disorder type II, YMRS = Young Mania Rating Scale | | | | | | | | | | | | | | |  |  |  |

| **Peak MNI coordinate** | ***Z*** | **P** | **Voxels** | **Brodmann areas** | **Regions** |
| --- | --- | --- | --- | --- | --- |
| **Major Depression<Healthy Controls** | | | | | |
| -66,-16,-12 | 2.64 | 0.002 | 19 | 21 | Left middle temporal gyrus |
| -30,-72,44 | 2.51 | 0.003 | 18 | 7 | Left inferior parietal lobule |
| -36,-34,-18 | 2.74 | 0.001 | 13 | 37 | Left fusiform gyrus |
| -4,-44,-22 | 2.46 | 0.004 | 13 | - | Cerebellar vermis |
| **Major Depression > Healthy Controls** | | | | | |
| -10,-72,0 | -1.04 | <0.001 | 207 | 18 | Left lingual gyrus |
| -8,-52,18 | -1.06 | <0.001 | 197 | 30 | Left precuneus |
| -48,-50,-40 | -1.01 | <0.001 | 129 | - | Left cerebellum, crus I |
| **Table S3.** Clusters showing differences between major depression and controls that did not meet our criteria for robustness | | | | | |

| **Peak MNI coordinate** | ***Z*** | **P** | **Voxels** | **Brodmann areas** | **Regions** |
| --- | --- | --- | --- | --- | --- |
| -46,4,4 | 1.82 | <0.001 | 436 | 48 | Left insula |
| 2,34,-16 | 1.33 | <0.001 | 248 | 11 | Left gyrus rectus, medial orbitofrontal cortex |
| 4,-2,42 | 1.15 | <0.001 | 203 | 23, 24 | Right midcingulate area |
| 22,2,-18 | 1.33 | <0.001 | 207 | 34 | Right amygdala |
| 52,6,-20 | 1.23 | <0.001 | 173 | 21 | Right temporal pole, middle temporal gyrus |
| -22,-16,-14 | 1.50 | <0.001 | 51 | 35 | Left hippocampus |
| -26,20,60 | 1.18 | <0.001 | 42 | 8 | Left middle frontal gyrus |
| **Table S4.** Clusters showing significant between study heterogeneity in major depression | | | | | |

| **Peak MNI coordinate** | ***Z*** | **P** | **Voxels** | **Brodmann areas** | **Regions** |
| --- | --- | --- | --- | --- | --- |
| **Bipolar Disorder<Healthy Controls** | | | | | |
| -62,-60,-10 | 2.71 | 0.002 | 20 | 37 | Left inferior temporal gyrus |
| 36,60,6 | 2.64 | 0.002 | 13 | 10 | Right middle frontal gyrus |
| **Bipolar Disorder>Healthy Controls** | | | | | |
| -14,-60,-44 | -1.21 | 0.003 | 23 | - | Left cerebellum, hemispheric lobule VIII |
| 4,-14,-22 | -1.33 | 0.002 | 16 | - | Right pons |
| -14,-30,-34 | -1.39 | 0.001 | 19 | - | Middle cerebellar peduncles |
| 4,-38,-18 | -1.17 | 0.003 | 20 | - | Cerebellar vermis |
| 42,-56,6 | -1.34 | 0.002 | 20 | 37 | Right middle temporal gyrus |
| -10,46,-30 | -1.38 | 0.001 | 11 | 11 | Left orbitofrontal cortex |
| -32,-54,38 | -1.22 | 0.003 | 11 | 40 | Left inferior parietal gyrus |
| **Table S5.** Clusters showing differences between bipolar disorder and controls did not meet our criteria for robustness | | | | | |

| **Peak MNI coordinate** | ***Z*** | **P** | **Voxels** | **Brodmann areas** | **Regions** |
| --- | --- | --- | --- | --- | --- |
| 8, 12, 12 | 5.88 | <0.001 | 657 | 25 | Right caudate nucleus |
| -46,38,-14 | 4.42 | <0.001 | 425 | 47 | Left inferior frontal gyrus |
| 30,-36,-18 | 3.297 | 0.001 | 169 | 37 | Right fusiform gyrus |
| 48,36,-12 | 3.94 | <0.001 | 119 | 47 | Right inferior frontal gyrus |
| -58,-66,-12 | 3.33 | <0.001 | 61 | 37 | Left inferior occipital gyrus |
| 6,18,22 | 2.82 | 0.002 | 55 | 24 | Right anterior cingulate cortex |
| 50,16,2 | 2.77 | 0.002 | 33 | 45, 48 | Right inferior frontal gyrus |
| 62,-20,-4 | 2.72 | 0.003 | 30 | 21, 22 | Right superior temporal gyrus, middle temporal gyrus |
| 6,-14,10 | 3.03 | 0.002 | 30 | - | Right thalamus |
| -20, -22, -22 | 2.89 | 0.002 | 28 | 30 | Left parahippocampal gyrus |
| -42, -46, 46 | 2.60 | 0.003 | 24 | 40 | Left inferior parietal gyrus |
| -52, 34, 24 | 3.39 | 0.001 | 18 | 45 | Left inferior frontal gyrus, pars triangularis |
| **Table S6.** Clusters showing significant between study heterogeneity in bipolar disorder | | | | | |

**Supplementary figures**

14951 results from literature search

340 selected for full text review

84 included in analyses

256 excluded (ROI approach, sample overlap, no coordinates reported, did not use an adult sample, shared sample with another study, did not compare with a healthy group, comorbid neurological illness, did not use VBM analysis)

14169 excluded based on title and abstract review (not relevant topic, duplicate search results, not VBM, ROI approach, did not use an adult sample, comorbid conditions)

Results from 2 unpublished studies acquired from authors

5 studies identified from review of reference lists

**Figure S1**. Literature search


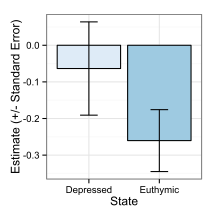

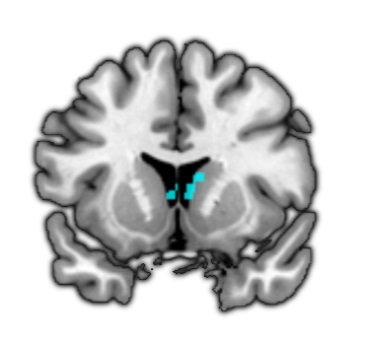


**A**

**B**


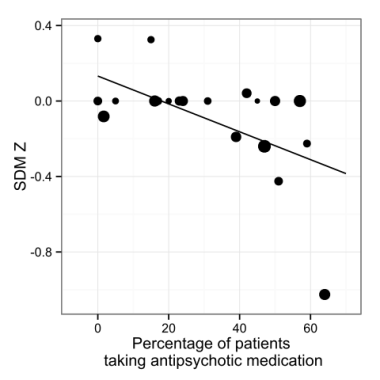

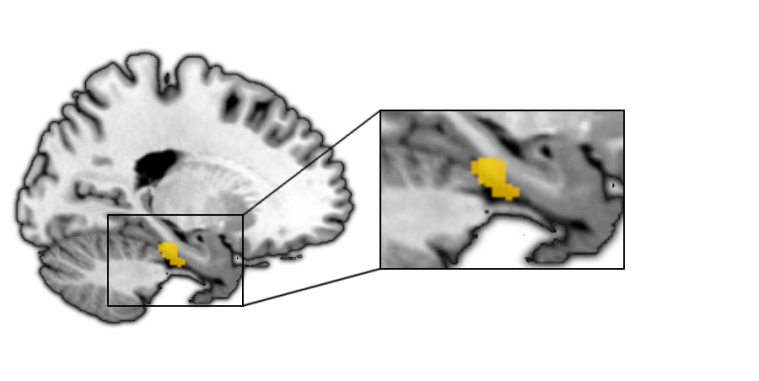


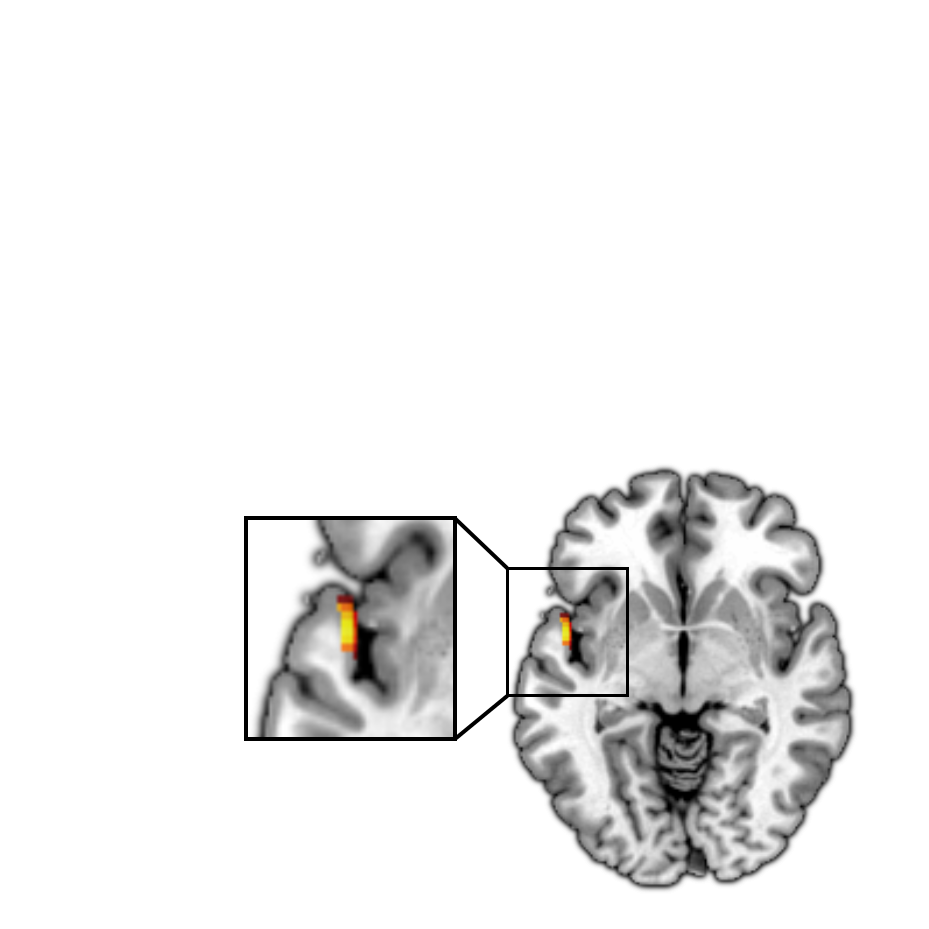

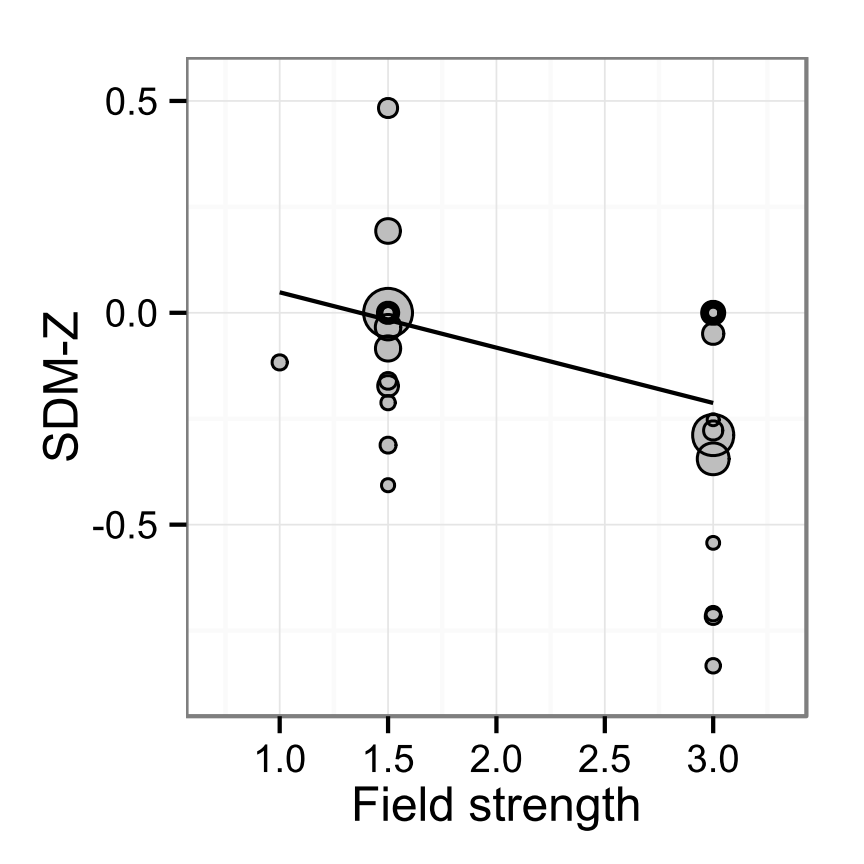


**C**


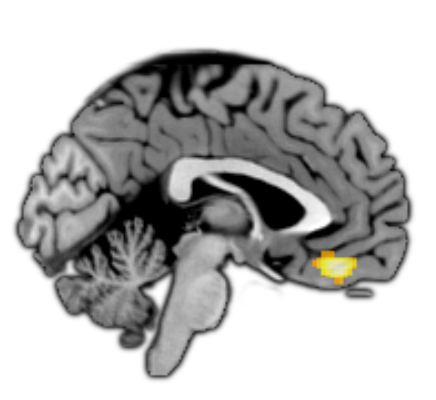

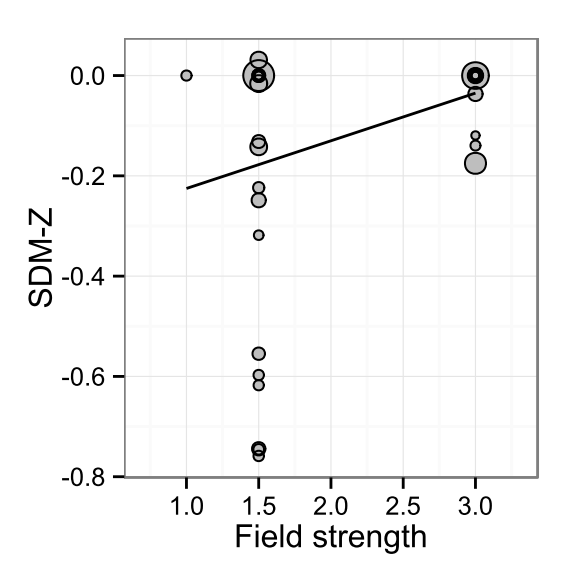


**D**

**Figure S2.** A) Results of mood state comparison in bipolar disorder.B) Results of meta-regression with antipsychotic medication load in bipolar disorder. C & D) Results of meta-regression with scanner field strength in major depression.

**Supplementary references**

1 Thompson SG, Higgins JPT. How should meta-regression analyses be undertaken and interpreted? *Stat Med* 2002; **21**: 1559–1573.

2 Radua J, Mataix-Cols D, Phillips ML, El-Hage W, Kronhaus DM, Cardoner N *et al.* A new meta-analytic method for neuroimaging studies that combines reported peak coordinates and statistical parametric maps. *Eur Psychiatry* 2012; **27**: 605–611.

3 Radua J, Romeo M, Mataix-Cols D, Fusar-Poli P. A General Approach for Combining Voxel-Based Meta-Analyses Conducted in Different Neuroimaging Modalities. *Curr Med Chem* 2013; **20**: 462–466.

4 Abe O, Yamasue H, Kasai K, Yamada H, Aoki S, Inoue H *et al.* Voxel-based analyses of gray/white matter volume and diffusion tensor data in major depression. *Psychiatry Res Neuroimaging* 2010; **181**: 64–70.

5 Amico F, Meisenzahl E, Koutsouleris N, Reiser M, Möller H-J, Frodl T. Structural MRI correlates for vulnerability and resilience to major depressive disorder. *J Psychiatry Neurosci JPN* 2011; **36**: 15–22.

6 Arnone D, McKie S, Elliott R, Juhasz G, Thomas EJ, Downey D *et al.* State-dependent changes in hippocampal grey matter in depression. *Mol Psychiatry* 2012. doi:10.1038/mp.2012.150.

7 Bergouignan L, Chupin M, Czechowska Y, Kinkingnéhun S, Lemogne C, Le Bastard G *et al.* Can voxel based morphometry, manual segmentation and automated segmentation equally detect hippocampal volume differences in acute depression? *NeuroImage* 2009; **45**: 29–37.

8 Chaney A, Carballedo A, Amico F, Fagan A, Skokauskas N, Meaney J *et al.* Effect of childhood maltreatment on brain structure in adult patients with major depressive disorder and healthy participants. *J Psychiatry Neurosci JPN* 2014; **39**: 50–59.

9 Cheng Y, Xu J, Chai P, Li H, Luo C, Yang T *et al.* Brain volume alteration and the correlations with the clinical characteristics in drug-naïve first-episode MDD patients: A voxel-based morphometry study.*Neurosci Lett* 2010; **480**: 30–34.

10 de Azevedo-Marques Périco C, Duran FLS, Zanetti MV, Santos LC, Murray RM, Scazufca M *et al.*A population-based morphometric MRI study in patients with first-episode psychotic bipolar disorder: comparison with geographically matched healthy controls and major depressive disorder subjects.*Bipolar Disord* 2011; **13**: 28–40.

11 Grieve SM, Korgaonkar MS, Koslow SH, Gordon E, Williams LM. Widespread reductions in gray matter volume in depression.*NeuroImageClin* 2013; **3**: 332–339.

12 Guo W, Liu F, Yu M, Zhang J, Zhang Z, Liu J *et al.* Functional and anatomical brain deficits in drug-naive major depressive disorder. *ProgNeuropsychopharmacolBiol Psychiatry* 2014; **54**: 1–6.

13 Inkster B, Rao AW, Ridler K, Nichols TE, Saemann PG, Auer DP *et al.* Structural Brain Changes in Patients with Recurrent Major Depressive Disorder Presenting with Anxiety Symptoms. *J Neuroimaging* 2011; **21**: 375–382.

14 Jia Z, Huang X, Wu Q, Zhang T, Lui S, Zhang J *et al.* High-Field Magnetic Resonance Imaging of Suicidality in Patients With Major Depressive Disorder. *Am J Psychiatry* 2010; **167**: 1381–1390.

15 Kim MJ, Hamilton JP, Gotlib IH.Reduced caudate gray matter volume in women with major depressive disorder.*Psychiatry Res Neuroimaging* 2008; **164**: 114–122.

16 Klauser P, Fornito A, Lorenzetti V, Davey CG, Dwyer DB, Allen NB *et al.*Cortico-limbic network abnormalities in individuals with current and past major depressive disorder. *J Affect Disord* 2015; **173**: 45–52.

17 Kong L, Wu F, Tang Y, Ren L, Kong D, Liu Y *et al.* Frontal-Subcortical Volumetric Deficits in Single Episode, Medication-Naïve Depressed Patients and the Effects of 8 Weeks Fluoxetine Treatment: A VBM-DARTEL Study. *PLoS ONE* 2014;**9**: e79055.

18 Kroes MCW, Rugg MD, Whalley MG, Brewin CR. Structural brain abnormalities common to posttraumatic stress disorder and depression.*J Psychiatry Neurosci JPN* 2011; **36**: 256–265.

19 Lai C-H, Hsu Y-Y, Wu Y-T. First episode drug-naïve major depressive disorder with panic disorder: Gray matter deficits in limbic and default network structures. *EurNeuropsychopharmacol* 2010; **20**: 676–682.

20 Lai C-H, Wu Y-T. Frontal-insula gray matter deficits in first-episode medication-naïve patients with major depressive disorder.*J Affect Disord* 2014; **160**: 74–79.

21 Lee H-Y, Tae WS, Yoon H-K, Lee B-T, Paik J-W, Son K-R *et al.* Demonstration of decreased gray matter concentration in the midbrain encompassing the dorsal raphe nucleus and the limbic subcortical regions in major depressive disorder: An optimized voxel-based morphometry study.*J Affect Disord* 2011; **133**: 128–136.

22 Leung K-K, Lee TMC, Wong MMC, Li LSW, Yip PSF, Khong P-L. Neural correlates of attention biases of people with major depressive disorder: a voxel-based morphometric study.*Psychol Med* 2009; **39**: 1097–1106.

23 Ma C, Ding J, Li J, Guo W, Long Z, Liu F *et al.* Resting-State Functional Connectivity Bias of Middle Temporal Gyrus and Caudate with Altered Gray Matter Volume in Major Depression. *PLoS ONE* 2012;**7**: e45263.

24 Legge RM, Sendi S, Cole JH, Cohen-Woods S, Costafreda SG, Simmons A *et al.* Modulatory effects of brain-derived neurotrophic factor Val66Met polymorphism on prefrontal regions in major depressive disorder. *Br J Psychiatry* 2015; **206**: 379–384.

25 Machino A, Kunisato Y, Matsumoto T, Yoshimura S, Ueda K, Yamawaki Y *et al.* Possible involvement of rumination in gray matter abnormalities in persistent symptoms of major depression: An exploratory magnetic resonance imaging voxel-based morphometry study.*J Affect Disord* 2014; **168**: 229–235.

26 Modinos G, Allen P, Frascarelli M, Tognin S, Valmaggia L, Xenaki L *et al.* Are we really mapping psychosis risk? Neuroanatomical signature of affective disorders in subjects at ultra high risk.*Psychol Med* 2014; **44**: 3491–3501.

27 Nakano M, Matsuo K, Nakashima M, Matsubara T, Harada K, Egashira K *et al.*Gray matter volume and rapid decision-making in major depressive disorder. *ProgNeuropsychopharmacolBiol Psychiatry* 2014; **48**: 51–56.

28 Peng J, Liu J, Nie B, Li Y, Shan B, Wang G *et al.* Cerebral and cerebellar gray matter reduction in first-episode patients with major depressive disorder: A voxel-based morphometry study. *Eur J Radiol* 2011; **80**: 395–399.

29 Peng H, Wu K, Li J, Qi H, Guo S, Chi M *et al.* Increased suicide attempts in young depressed patients with abnormal temporal–parietal–limbic gray matter volume. *J Affect Disord* 2014; **165**: 69–73.

30 Redlich R, Almeida JJR, Grotegerd D, Opel N, Kugel H, Heindel W *et al.* Brain morphometric biomarkers distinguishing unipolar and bipolar depression. A voxel-based morphometry-pattern classification approach.*JAMA Psychiatry* 2014; **71**: 1222–1230.

31 Rodríguez-Cano E, Sarró S, Monté GC, Maristany T, Salvador R, McKenna PJ *et al.* Evidence for structural and functional abnormality in the subgenual anterior cingulate cortex in major depressive disorder. *Psychol Med* 2014; **44**: 3263–3273.

32 Salvadore G, Nugent AC, Lemaitre H, Luckenbaugh DA, Tinsley R, Cannon DM *et al.* Prefrontal cortical abnormalities in currently depressed versus currently remitted patients with major depressive disorder. *NeuroImage* 2011; **54**: 2643–2651.

33 Scheuerecker J, Meisenzahl EM, Koutsouleris N, Roesner M, Schöpf V, Linn J *et al.* Orbitofrontal volume reductions during emotion recognition in patients with major depression. *J Psychiatry Neurosci JPN* 2010; **35**: 311–320.

34 Serra-Blasco M, Portella MJ, Gómez-Ansón B, Diego-Adeliño J de, Vives-Gilabert Y, Puigdemont D *et al.* Effects of illness duration and treatment resistance on grey matter abnormalities in major depression. *Br J Psychiatry* 2013; **202**: 434–440.

35 Shah PJ, Ebmeier KP, Glabus MF, Goodwin GM. Cortical grey matter reductions associated with treatment-resistant chronic unipolar depression. Controlled magnetic resonance imaging study.*Br J Psychiatry* 1998; **172**: 527–532.

36 Soriano-Mas C, Hernández-Ribas R, Pujol J, Urretavizcaya M, Deus J, Harrison BJ *et al.* Cross-Sectional and Longitudinal Assessment of Structural Brain Alterations in Melancholic Depression. *Biol Psychiatry* 2011; **69**: 318–325.

37 Sprengelmeyer R, Steele JD, Mwangi B, Kumar P, Christmas D, Milders M *et al.*The insular cortex and the neuroanatomy of major depression.*J Affect Disord* 2011; **133**: 120–127.

38 Stratmann M, Konrad C, Kugel H, Krug A, Schöning S, Ohrmann P *et al.* Insular and Hippocampal Gray Matter Volume Reductions in Patients with Major Depressive Disorder. *PLoS ONE* 2014;**9**: e102692.

39 Treadway MT, Grant MM, Ding Z, Hollon SD, Gore JC, Shelton RC. Early Adverse Events, HPA Activity and Rostral Anterior Cingulate Volume in MDD.*PLoS ONE* 2009;**4**: e4887.

40 Wagner G, Koch K, Schachtzabel C, Schultz CC, Sauer H, Schlösser RG. Structural brain alterations in patients with major depressive disorder and high risk for suicide: Evidence for a distinct neurobiological entity?*NeuroImage* 2011; **54**: 1607–1614.

41 Wang L, Dai W, Su Y, Wang G, Tan Y, Jin Z *et al.* Amplitude of Low-Frequency Oscillations in First-Episode, Treatment-Naive Patients with Major Depressive Disorder: A Resting-State Functional MRI Study. *PLoS ONE* 2012;**7**: e48658.

42 Wang L, Wang T, Liu S, Liang Z, Meng Y, Xiong X *et al.* Cerebral anatomical changes in female asthma patients with and without depression compared to healthy controls and patients with depression. *J Asthma* 2014; **51**: 927–933.

43 Yoshikawa E, Matsuoka Y, Yamasue H, Inagaki M, Nakano T, Akechi T *et al.* Prefrontal Cortex and Amygdala Volume in First Minor or Major Depressive Episode After Cancer Diagnosis. *Biol Psychiatry* 2006; **59**: 707–712.

44 Zou K, Deng W, Li T, Zhang B, Jiang L, Huang C *et al.* Changes of Brain Morphometry in First-Episode, Drug-Naïve, Non–Late-Life Adult Patients with Major Depression: An Optimized Voxel-Based Morphometry Study. *Biol Psychiatry* 2010; **67**: 186–188.

45 Adler CM, Levine AD, DelBello MP, Strakowski SM. Changes in Gray Matter Volume in Patients with Bipolar Disorder. *Biol Psychiatry* 2005; **58**: 151–157.

46 Almeida JRC, Akkal D, Hassel S, Travis MJ, Banihashemi L, Kerr N *et al.* Reduced gray matter volume in ventral prefrontal cortex but not amygdala in bipolar disorder: Significant effects of gender and trait anxiety. *Psychiatry Res Neuroimaging* 2009; **171**: 54–68.

47 Ambrosi E, Rossi-Espagnet MC, Kotzalidis GD, Comparelli A, Del Casale A, Carducci F *et al.* Structural brain alterations in bipolar disorder II: A combined voxel-based morphometry (VBM) and diffusion tensor imaging (DTI) study. *J Affect Disord* 2013; **150**: 610–615.

48 Brown GG, Lee J-S, Strigo IA, Caligiuri MP, Meloy MJ, Lohr J. Voxel-based morphometry of patients with schizophrenia or bipolar I disorder: A matched control study. *Psychiatry Res Neuroimaging* 2011; **194**: 149–156.

49 Bruno SD, Barker GJ, Cercignani M, Symms M, Ron MA. A study of bipolar disorder using magnetization transfer imaging and voxel-based morphometry. *Brain* 2004; **127**: 2433–2440.

50 Chen X, Wen W, Malhi GS, Ivanovski B, Sachdev PS. Regional Gray Matter Changes in Bipolar Disorder: A Voxel-Based Morphometric Study. *Aust N Z J Psychiatry* 2007; **41**: 327–336.

51 Chen Z, Cui L, Li M, Jiang L, Deng W, Ma X *et al.* Voxel based morphometric and diffusion tensor imaging analysis in male bipolar patients with first-episode mania. *ProgNeuropsychopharmacolBiol Psychiatry* 2012; **36**: 231–238.

52 Cui L, Li M, Deng W, Guo W, Ma X, Huang C *et al.* Overlapping clusters of gray matter deficits in paranoid schizophrenia and psychotic bipolar mania with family history. *Neurosci Lett* 2011; **489**: 94–98.

53 Doris A, Belton E, Ebmeier KP, Glabus MF, Marshall I. Reduction of cingulate gray matter density in poor outcome bipolar illness. *Psychiatry Res Neuroimaging* 2004; **130**: 153–159.

54 Eker C, Simsek F, Yılmazer EE, Kitis O, Cinar C, Eker OD *et al.* Brain regions associated with risk and resistance for bipolar I disorder: a voxel-based MRI study of patients with bipolar disorder and their healthy siblings. *Bipolar Disord* 2014; **16**: 249–261.

55 Emsell L, Langan C, Van Hecke W, Barker GJ, Leemans A, Sunaert S *et al.* White matter differences in euthymic bipolar I disorder: a combined magnetic resonance imaging and diffusion tensor imaging voxel-based study. *Bipolar Disord* 2013; **15**: 365–376.

56 Ha TH, Ha K, Kim JH, Choi JE. Regional brain gray matter abnormalities in patients with bipolar II disorder: A comparison study with bipolar I patients and healthy controls. *Neurosci Lett* 2009; **456**: 44–48.

57 Haldane M, Cunningham G, Androutsos C, Frangou S. Structural brain correlates of response inhibition in Bipolar Disorder I. *J Psychopharmacol (Oxf)* 2008; 22: 138–143.

58 Kempton MJ. *Unpublished Manuscript* 2015.

59 Li M, Cui L, Deng W, Ma X, Huang C, Jiang L *et al.* Voxel-based morphometric analysis on the volume of gray matter in bipolar I disorder. *Psychiatry Res Neuroimaging* 2011; **191**: 92–97.

60 Lochhead RA, Parsey RV, Oquendo MA, Mann JJ. Regional brain gray matter volume differences in patients with bipolar disorder as assessed by optimized voxel-based morphometry. *Biol Psychiatry* 2004; **55**: 1154–1162.

61 Lyoo IK, Kim MJ, Stoll AL, Demopulos CM, Parow AM, Dager SR *et al.* Frontal lobe gray matter density decreases in bipolar I disorder. *Biol Psychiatry* 2004; **55**: 648–651.

62 McDonald C, Bullmore E, Sham P, Chitnis X, Suckling J, MacCabe J *et al.* Regional volume deviations of brain structure in schizophrenia and psychotic bipolar disorder Computational morphometry study. *Br J Psychiatry* 2005; **186**: 369–377.

63 McIntosh AM, Job DE, Moorhead TWJ, Harrison LK, Forrester K, Lawrie SM *et al.* Voxel-based morphometry of patients with schizophrenia or bipolar disorder and their unaffected relatives. *Biol Psychiatry* 2004; **56**: 544–552.

64 Molina V, Galindo G, Cortés B, Herrera AGS de, Ledo A, Sanz J *et al.* Different gray matter patterns in chronic schizophrenia and chronic bipolar disorder patients identified using voxel-based morphometry. *Eur Arch Psychiatry ClinNeurosci* 2011; **261**: 313–322.

65 Narita K, Suda M, Takei Y, Aoyama Y, Majima T, Kameyama M *et al.* Volume reduction of ventromedial prefrontal cortex in bipolar II patients with rapid cycling: A voxel-based morphometric study.*ProgNeuropsychopharmacolBiol Psychiatry* 2011; **35**: 439–445.

66 Nugent AC, Milham MP, Bain EE, Mah L, Cannon DM, Marrett S *et al.* Cortical abnormalities in bipolar disorder investigated with MRI and voxel-based morphometry. *NeuroImage* 2006; **30**: 485–497.

67 Rocha-Rego V, Jogia J, Marquand AF, Mourao-Miranda J, Simmons A, Frangou S. Examination of the predictive value of structural magnetic resonance scans in bipolar disorder: a pattern classification approach. *Psychol Med* 2013; **FirstView**: 1–14.

68 Scherk H, Kemmer C, Usher J, Reith W, Falkai P, Gruber O. No change to grey and white matter volumes in bipolar I disorder patients. *Eur Arch Psychiatry ClinNeurosci* 2008; **258**: 345–349.

69 Shepherd AM, Quidé Y, Laurens KR, O’Reilly N, Rowland JE, Mitchell PB *et al.* Shared intermediate phenotypes for schizophrenia and bipolar disorder: neuroanatomical features of subtypes distinguished by executive dysfunction. *J Psychiatry Neurosci JPN* 2014; **39**: 130283.

70 Stanfield AC, Moorhead TWJ, Job DE, McKirdy J, Sussmann JE, Hall J *et al.* Structural abnormalities of ventrolateral and orbitofrontal cortex in patients with familial bipolar disorder. *Bipolar Disord* 2009; **11**: 135–144.

71 Tang L-R, Liu C-H, Jing B, Ma X, Li H-Y, Zhang Y *et al.* Voxel-based morphometry study of the insular cortex in bipolar depression.*Psychiatry Res Neuroimaging* 2014; **224**: 89–95.

72 Yatham LN, Lyoo IK, Liddle P, Renshaw PF, Wan D, Lam RW *et al.*A magnetic resonance imaging study of mood stabilizer- and neuroleptic-naïve first-episode mania.*Bipolar Disord* 2007; **9**: 693–697.

73 Yüksel C, McCarthy J, Shinn A, Pfaff DL, Baker JT, Heckers S *et al.*Gray matter volume in schizophrenia and bipolar disorder with psychotic features. *Schizophr Res* 2012; **138**: 177–182.
